# Supplementary material for: Directed Evolution of Near-Infrared Serotonin Nanosensors with Machine Learning-Based Screening
Source: Nanomaterials (Basel). 2024 Jan 23;14(3):247. doi: 10.3390/nano14030247 (PMC10856788; doi:10.3390/nano14030247)
Supplement: Supplementary file 1 [file nanomaterials-14-00247-s001.zip › nanomaterials-2836621-supplementary.pdf]

Supplementary Materials

Directed Evolution of Near-Infrared Serotonin Nanosensors with Machine Learning-Based Screening

Seonghyeon An [1,†], Yeongjoo Suh [1,†], Payam Kelich [2], Dakyeon Lee [1,3], Lela Vukovic [2] and Sanghwa Jeong [1,*]

[1] Department of Biomedical Convergence Engineering, Pusan National University, Yangsan 50612, Republic of Korea
[2] Department of Chemistry and Biochemistry, University of Texas at El Paso, El Paso, TX 79968, USA
[3] Department of Chemistry, Pohang University of Science and Technology (POSTECH), Pohang 37673, Republic of Korea
* Correspondence: sanghwa.jeong@pusan.ac.kr; Tel.: +82-51-510-8539
† These authors contributed equally to this work.

a.

Sequence ID	DNA sequence (from 5' to 3')	Predicted $\Delta F/F_0$	Sequence ID	DNA sequence (from 5' to 3')	Predicted $\Delta F/F_0$
1	CCCCCCAGCCCAACACAACCAACTCCCCCC	1.533	11	CCCCCCAGCCCAACACCACCAGCTCCCCCC	1.456
2	CCCCCCAGCCCTACACAACCAGCTCCCCCC	1.491	12	CCCCCCAGCCCGACACAACCAACTCCCCCC	1.451
3	CCCCCCAGCCCTACACAACCACCTCCCCCC	1.480	13	CCCCCCAGCCCTACACAACCAACTCCCCCC	1.449
4	CCCCCCAGCCCTACACAACCAACGCCCCCC	1.479	14	CCCCCCAGCACTACACAACCAACTCCCCCC	1.448
5	CCCCCCAGCCCTACACATCCAACTCCCCCC	1.469	15	CCCCCCAGCCCATCACAACCAACGCCCCCC	1.447
6	CCCCCCAGCCCTACCCAACCAACTCCCCCC	1.468	16	CCCCCCAGCCCTAAACAACCAACTCCCCCC	1.444
7	CCCCCCAACCCTACACAACCAACTCCCCCC	1.467	17	CCCCCCAGCCCATCACAACCAGCTCCCCCC	1.442
8	CCCCCCAGGCCTACACAACCAACTCCCCCC	1.466	18	CCCCCCAGCCCTACACAACAAACTCCCCCC	1.441
9	CCCCCCAGCCCAACACCACCACCTCCCCCC	1.463	19	CCCCCCAGCCCAACACGACCAACTCCCCCC	1.440
10	CCCCCCAGCCCTAGACAACCAACTCCCCCC	1.458	20	CCCCCCAGCCCTACACAACCAACCCCCCCC	1.439

b.

Sequence ID	DNA sequence (from 5' to 3')	Predicted $\Delta F/F_0$	Sequence ID	DNA sequence (from 5' to 3')	Predicted $\Delta F/F_0$
1	CCCCCCAGCCCAACACAACCAACTCCCCCC	1.533	11	CCCCCCAGCCCAACACCACCAGCTCCCCCC	1.456
2	CCCCCCAGCCCTACACAACCAGCTCCCCCC	1.491	12	CCCCCCAGCCCGACACAACCAACTCCCCCC	1.451
3	CCCCCCAGCCCTACACAACCACCTCCCCCC	1.480	13	CCCCCCAGCCCTACACAACCAACTCCCCCC	1.449
4	CCCCCCAGCCCTACACAACCAACGCCCCCC	1.479	14	CCCCCCAGCACTACACAACCAACTCCCCCC	1.448
5	CCCCCCAGCCCTACACATCCAACTCCCCCC	1.469	15	CCCCCCAGCCCATCACAACCAACGCCCCCC	1.447
6	CCCCCCAGCCCTACCCAACCAACTCCCCCC	1.468	16	CCCCCCAGCCCTAAACAACCAACTCCCCCC	1.444
7	CCCCCCAACCCTACACAACCAACTCCCCCC	1.467	17	CCCCCCAGCCCATCACAACCAGCTCCCCCC	1.442
8	CCCCCCAGGCCTACACAACCAACTCCCCCC	1.466	18	CCCCCCAGCCCTACACAACAAACTCCCCCC	1.441
9	CCCCCCAGCCCAACACCACCACCTCCCCCC	1.463	19	CCCCCCAGCCCAACACGACCAACTCCCCCC	1.440
10	CCCCCCAGCCCTAGACAACCAACTCCCCCC	1.458	20	CCCCCCAGCCCTACACAACCAACCCCCCCC	1.439

c.

Sequence ID	DNA sequence (from 5' to 3')	Predicted $\Delta F/F_0$	Sequence ID	DNA sequence (from 5' to 3')	Predicted $\Delta F/F_0$
1	CCCCCCAGCCCAACACAACCAACTCCCCCC	1.533	11	CCCCCCAGCCCAACACCACCAGCTCCCCCC	1.456
2	CCCCCCAGCCCTACACAACCAGCTCCCCCC	1.491	12	CCCCCCAGCCCGACACAACCAACTCCCCCC	1.451
3	CCCCCCAGCCCTACACAACCACCTCCCCCC	1.480	13	CCCCCCAGCCCTACACAACCAACTCCCCCC	1.449
4	CCCCCCAGCCCTACACAACCAACGCCCCCC	1.479	14	CCCCCCAGCACTACACAACCAACTCCCCCC	1.448
5	CCCCCCAGCCCTACACATCCAACTCCCCCC	1.469	15	CCCCCCAGCCCATCACAACCAACGCCCCCC	1.447
6	CCCCCCAGCCCTACCCAACCAACTCCCCCC	1.468	16	CCCCCCAGCCCTAAACAACCAACTCCCCCC	1.444
7	CCCCCCAACCCTACACAACCAACTCCCCCC	1.467	17	CCCCCCAGCCCATCACAACCAGCTCCCCCC	1.442
8	CCCCCCAGGCCTACACAACCAACTCCCCCC	1.466	18	CCCCCCAGCCCTACACAACAAACTCCCCCC	1.441
9	CCCCCCAGCCCAACACCACCACCTCCCCCC	1.463	19	CCCCCCAGCCCAACACGACCAACTCCCCCC	1.440
10	CCCCCCAGCCCTAGACAACCAACTCCCCCC	1.458	20	CCCCCCAGCCCTACACAACCAACCCCCCCC	1.439

d.

Sequence ID	DNA sequence (from 5' to 3')	Predicted $\Delta F/F_0$	Sequence ID	DNA sequence (from 5' to 3')	Predicted $\Delta F/F_0$
1	CCCCCCAACACAACACAACCACCTCCCCCC	1.498	11	CCCCCCAGCACTACCCAACCACCCCCCCCC	1.395
2	CCCCCCAGCACAACACAACCACCGCCCCCC	1.482	12	CCCCCCAACACTACACAAGCACCTCCCCCC	1.394
3	CCCCCCAGCACAACACAACCACCCCCCCCC	1.455	13	CCCCCCAACACTACACAACCACCACCCCCC	1.394
4	CCCCCCAACACTACCCAACCACCTCCCCCC	1.453	14	CCCCCCGACACTACACAACCACCTCCCCCC	1.390
5	CCCCCCAGCACAACACAAGCACCTCCCCCC	1.443	15	CCCCCCAACACTACACGACCACCTCCCCCC	1.387
6	CCCCCCAACGCTACACAACCACCTCCCCCC	1.422	16	CCCCCCAGCGCTACACAACCACCGCCCCCC	1.381
7	CCCCCCAACACTACACAACCACCGCCCCCC	1.420	17	CCCCCCGGCACTACACAACCACCGCCCCCC	1.367
8	CCCCCCAGCACAACACTACCACCTCCCCCC	1.411	18	CCCCCCAGCACTACACGACCACCGCCCCCC	1.366
9	CCCCCCAGCACAACACCACCACCTCCCCCC	1.403	19	CCCCCCAGCACAACACCACCAGCTCCCCCC	1.362
10	CCCCCCAACACTACACAACCACCCCCCCCC	1.395	20	CCCCCCAGGACTACACCACCACCTCCCCCC	1.361

Figure S1. The table shows the top 20 sequences in each round of machine learning and the predicted machine learning results. The predicted $\Delta F/F_0$ is the predicted value of how well the sequence responds to serotonin at 1195 nm. For the original sequence in each round, the positions where 3 base mutations occurred and the results are coloured in red. **(a)** Table for round 1 of sensitivity, selectivity. **(b)** Table for round 2 of sensitivity. **(c)** Table for round 3 of sensitivity. **(d)** Table for round 2 of selectivity.

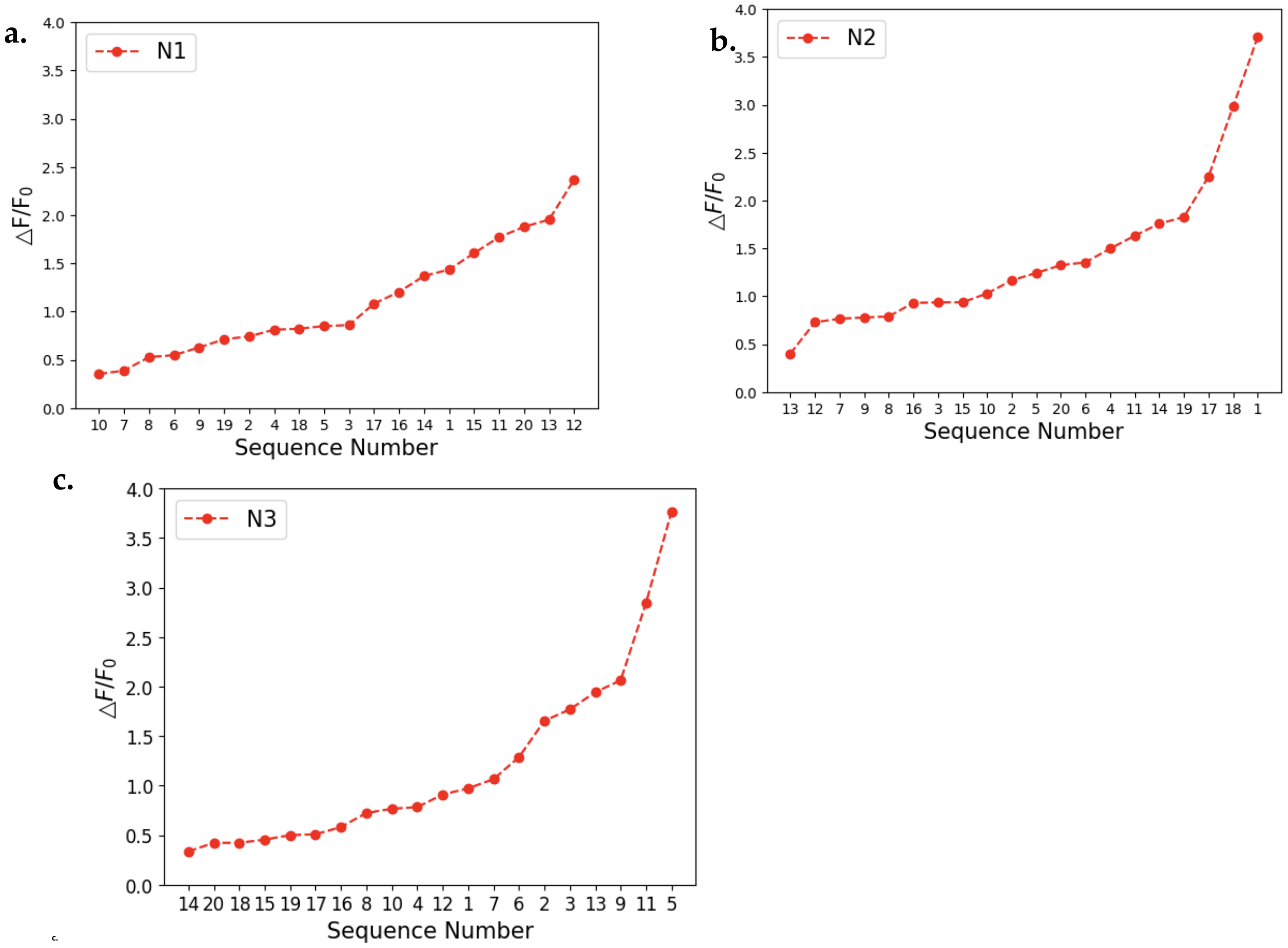

Figure S2. The $\Delta F/F_0$ values of the top 20 sequences for each round in sensitivity, sorted in ascending order. **(a)** The best performing sequence in round N1 is N1-12, with a $\Delta F/F_0$ of 2.366. **(b)** The best performing sequence N2-1 with a $\Delta F/F_0$ of 3.705. The second best performing sequence, N2-18, has a $\Delta F/F_0$ of 2.981, which is better than N1-12. **(c)** The best performing sequence N3-5 with a $\Delta F/F_0$ of 3.765.

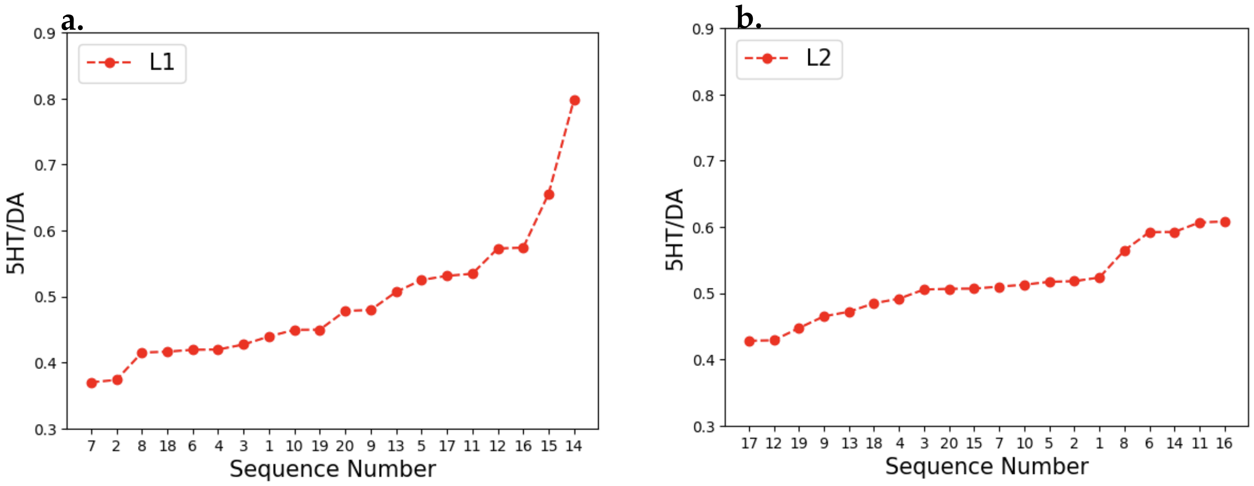

Figure S3. The 5HT/DA values of the top 20 sequences for each round in selectivity, sorted in ascending order. **(a)** The best performing sequence in the L1 round, L1-14, has a value of 0.799, while the second best performing sequence, L1-15, has a 5HT/DA value of 0.655. **(b)** The best performing sequence in the L2 round, L2-16, has a 5HT/DA value of 0.608. The 5HT/DA values of the remaining sequences are distributed between 0.4 and 0.6, and L2-16 has a value smaller than the 5HT/DA value of L1-15, which is 0.655, indicating that it was saturated in the L1 round.
